# LncRNA-PANDAR regulates the progression of thyroid carcinoma by targeting miR-637/KLK4

**DOI:** 10.7150/jca.55181

**Published:** 2021-08-08

**Authors:** Yi Qing, Qian Li, Ling-Yan Zhao, Ping Shi, Jin-Lu Shan, Wei Zhang

**Affiliations:** 1 Department of Oncology, Affiliated Hospital of Chengdu University, Chengdu 610081, People's Republic of China.; 2 Department of Oncology, Daping hospital, Army Medical University, Chongqing, 400042, China.; 3 Department of respiratory and critical care medicine, Guangyuan Central Hospital, Guangyuan City, Sichuan Province, 628000, China.; 4 Department of medical oncology, Sichuan Cancer Hospital & Institute, Sichuan Cancer Centre, School of Medicine, University of Electronic Science and Technology of China, Chengdu, 610041, China.

**Keywords:** Thyroid gland carcinoma, LncRNA PANDAR, MiR-637, KLK4, Progression

## Abstract

Thyroid gland carcinoma (TC) originates from follicular or parafollicular thyroid cells and is one of the most common endocrine organ malignancies. To explore the molecular mechanism by which long-chain non-coding RNAs regulate the growth and metastasis of thyroid gland carcinoma, in this study we focused on long non-coding RNAs (lncRNAs) that have been reported to be involved in tumorigenesis. We identified Promoter Region of CDKN 1A antisense DNA damage-activated RNA (PANDAR), which was positively correlated with thyroid gland carcinoma risk. PANDAR could promote thyroid gland carcinoma cell proliferation and metastasis. PANDAR negatively correlated with miR-637, and miR-637 overexpression suppressed thyroid gland carcinoma progression, which could be reversed by PANDAR. MiR-637 could target Kallikrein-related peptidases 4 (KLK4) to inhibit its expression, which was high in thyroid gland carcinoma. KLK4 inhibited cell progression in thyroid gland carcinoma cells. Knockdown of PANDAR expression inhibited cancer progression in nude mice. Overall, PANDAR can suppress miR-637 and induce KLK4 to regulate invasion and migration in thyroid gland carcinoma. Additionally, we identified miR-637 as a target of PANDAR in thyroid gland carcinoma, and PANDAR can be used as a novel therapeutic target for the treatment of thyroid gland carcinoma.

## Introduction

Thyroid gland carcinoma (TC) originates from follicular or parafollicular thyroid cells and is one of the most common endocrine organ malignancies. Its morbidity and mortality have steadily increased over the years 1. Previous studies have shown that radiation therapy, chemotherapy and surgery are common treatments for thyroid gland carcinoma, but none are very effective 2, 3. Some studies have shown that in addition to environmental and genetic susceptibility factors, epigenetic changes may play a key role in the occurrence and development of various tumours, including thyroid carcinoma 4, 5.

As important participants in tumour biology, long non-coding RNAs (lncRNAs) are involved in many regulatory functions, including regulation of apoptosis and invasion and induction of reprogramming of pluripotent stem cells 6, 7. Abnormal expression of a number of lncRNAs is often associated with cancer pathogenesis 8, 9. LncRNA research provides new ideas for the study of cancer genetics and molecular mechanisms.

PANDAR, a novel non-coding RNA located in the promoter region of CDKN 1A antisense DNA damage-activated RNA, is located at chromosome 6p21.2. PANDAR is an oncogenic in bladder cancer, promoting cell proliferation, migration, and apoptosis 10. In addition, Han et al. showed that low expression of the long-chain non-coding RNA PANDAR predicts poor prognosis in non-small cell lung cancer and affects apoptosis by modulating Bcl-2^11^. It has recently been reported that PANDAR is highly expressed in thyroid gland carcinoma and can promote tumour proliferation and inhibit apoptosis. However, how PANDAR regulates proliferation and apoptosis still needs to be studied.

miRNAs can participate in tumour progression by regulating the post-transcriptional levels of their target genes and may be involved in the regulation of the expression of many important tumour-associated genes and the activity of multiple signalling pathways 12. MiR-637 has been reported to be abnormally expressed in tumours, such as breast cancer, glioma, and prostate cancer, and is closely related to tumour proliferation and metastasis 13-16. At present, there is no research to explore its effect on thyroid gland carcinoma. Kallikrein-related peptidases (KLKs) are a class of secreted serine proteases with trypsin or chymotrypsin activity and are composed of 15 members (KLK1-15) 17. Recent studies have found that the KLK family plays an important role in tumour progression and is thought to be a key factor in regulating tumour cell proliferation, migration and invasion 18, 19. KLK4 is a member of this family, and previous studies have shown that together with KLK5, it can activate precursor hepatocyte growth factor activator indirectly, whereby precursor hepatocyte growth factor dispersing factors are transformed into mature and activated forms that promote the progression of a variety of tumours 19, 20. According to Obiezu 19, 21, overexpression of KLK4 induces proliferation and increases the risk of recurrence and death in patients with ovarian cancer. The results of these reports indicate that KLK4 may be a valuable biomarker in the diagnosis and treatment of ovarian cancer. However, other studies on thyroid gland carcinoma have rarely been reported.

In this study, we explored the expression of PANDAR in thyroid gland carcinoma tissues and thyroid gland carcinoma cell lines. In addition, the mechanism of action of PANDAR in the regulation of the proliferation and metastasis of thyroid gland carcinoma cells by miR-637 was examined *in vitro* and vivo.

## Materials and Methods

### Clinical information and cell culture

Paired samples information in Table 1, were obtained from patients examined and undergoing surgery at Department of Oncology, Affiliated Hospital of Chengdu University. Prior to the use of these clinical materials for research purposes, all patients provided informed consent and were approved by the Ethics Committee of the Affiliated Hospital of Chengdu University. The experimental protocol was approved by the Ethics Committee of Affiliated Hospital of Chengdu University.

The Nthy-ori3-1 cells purchased from the American Type Culture Collection and human thyroid gland carcinoma K-1, TPC-1, K1, FTC133 and XTC-1 cells were cultured in RPMI-1640 medium with 10% foetal bovine serum (FBS), in a humidified incubator containing 5% CO_2_ at 37 °C. The cells were grown in a single layer and routinely passaged when the cells were 90% confluent.

### SiRNA, miRNA and plasmid DNA transfection

A PANDAR-siRNA expression vector was constructed based on the full length wild-type lncRNA-PANDAR coding sequence of GeneChem Biotech (Shanghai, China). The target sequence used to construct the siRNA-PANDAR vector was antisense: PANDAR-1: 5'-GCAATCTACAACCTGTCTT-3' and PANDAR-1: 5'-TTTCGAACGGAACAGAGACUUAUACAGATT-3'. miR‐637 primer: Forward 5′‐ACACTCCAGCTGGGACTGGGGGCTTTCGGGCT‐3′, Reverse 5′‐CTCAACTGGTGTCGTGGAGTCGGCAATTCAGTTGAGACGCAGAG‐3′; miR‐637 mimics: Sense 5′‐ACUGGGGGCUUUCGGGCUCUGCGU‐3′, Antisense 5′‐GCAGAGCCCGAAAGCCCCCAGUUU‐3′; KLK4 Primer: Forward 5′-CCAGAGTACAACAGACCCTTGC‐3′, Reverse 5′-AGCACGGTAGGCATTCTGC‐3′; KLK4 siRNA: 5′-AATCCGTGTCCGAGTCTGAC‐3′ The siRNA vector without the PANDAR siRNA sequence (si-NC) was transfected into thyroid gland carcinoma cells as a control. Cells were seeded and grown in growth medium until the cell density reached 70%. Then, siRNA transfection was performed with Lipofectamine 2000 reagent based on the manufacturer's protocol (Invitrogen, USA). Cells were harvested 48 hours later for qRT-PCR and Western blot analysis.

### MTT assay cell viability

After 24 hours of transfection, cells were reseeded in 96-well plate at density 10^4^/well. MTT assay was performed using MTT assay kit (promega) according to the manufacturer's protocol.

### Transwell assay

The cells were transfected with lentivirus for treatment, and puromycin was used to select stable cell lines. The cells were seeded in the upper chamber with a medium containing 0.1% bovine serum albumin, and medium containing 30% FBS was placed in the lower chamber. Transwell assays were performed as described by Wei et al. 22. Three independent experiments were performed.

### Luciferase reporter assay

PANDAR wild-type and mutant firefly luciferase plasmids were constructed, and firefly luciferase plasmid, sea cucumber luciferase plasmid and miR-637 were co-transfected into 293T cells for 72 h, and 100 μl of special lysate was added to collect the lysate. For the product, 50 μl of the dissociated product was added to the white plate, 50 μl of firefly luciferin substrate was added, following 48 hours of incubation, cells were subjected to a luciferase reporter assay. Luciferase activity was measured using the dual-luciferase assay system according to the manufacturer's protocol (Promega, Madison, WI).

### Apoptosis assay

After transfection with siRNA, the cells were further cultured for 48 hours and the apoptosis was detected using Annexin V-FITC Apoptosis Detection Kit (BD, USA). Stained cells were analysed by FACSCalibur Flow Cytometer (BD Biosciences) 22.

### Western blot analysis

Total 30 µg of protein was separated by sodium dodecyl sulfate-polyacrylamide gel electrophoresis and transferred onto nitrocellulose membranes. The primary antibodies KLK4 (CST, USA, Cat.NO: #5471, 1:2000), β-actin (CST, Cat. NO.: #3700, 1:5000), Western blot analysis methods are as described in the references 22. The Western bands were quantified using Image J program.

### Animal experiments

All experimental procedures involving animals were in accordance with the Guide for the Care and Use of Laboratory Animals, and were performed according to the institutional ethical guidelines for animal experiments. All studies involving animals were approved by the Affiliated Hospital of Chengdu University. Female BALB/C nude mice (5/groups, 5 weeks old) were raised in the animal facility during the experiment procedures with free access to diet or water. Briefly, 5×10^6^ TPC-1 cells in 100 µl serum-free medium were injected subcutaneously (s.c.) into per mouse (right back). Seven animals were randomly divided into each study group. After 6 weeks after implantation, mice were sacrificed, and the xenograft tumour were analysed by standard histological examination.

### Statistical Analysis

Three independent experiments were performed. All data in this study were expressed as mean ± sd, and differences between groups were determined using analysis of variance (Anova) of SPSS version 18.0. p<0.05 was considered statistically significant.

## Results

### PANDAR expression is upregulated in thyroid gland carcinoma tissue and cell lines

**Figure 1:**
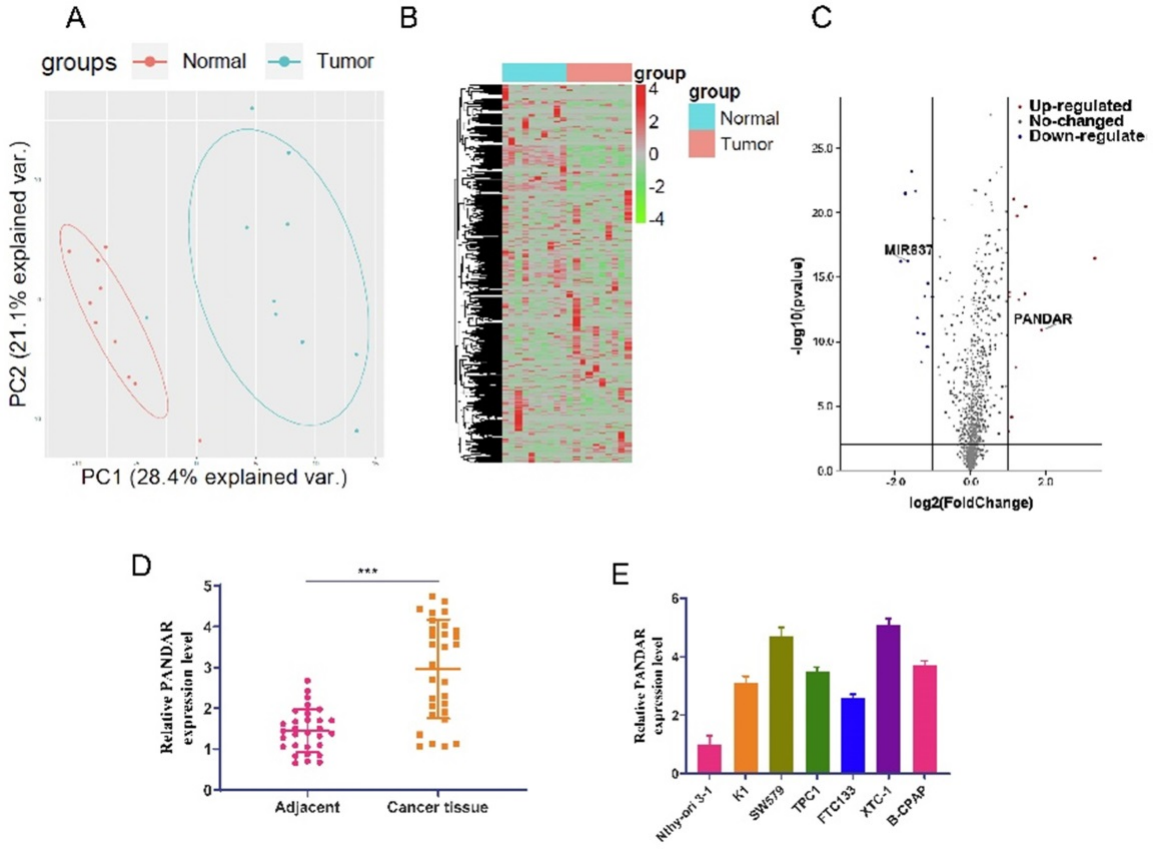
** LncRNA-PANDAR overexpression in thyroid gland carcinoma tissue and cell lines. (A)** 10 pairs of thyroid gland carcinoma and adjacent tissue sequencing results for bioinformatics analysis PCA analysis showed the difference between cancer and adjacent groups. **(B)** Heat map showing cancer adjacent groups all non-coding RNA expression; the RNA detection data is analysed by the R language toolkit. **(C)** Volcano plots analysis of non-coding RNA differences. The expression of LncRNA-PANDAR and miR-637 significant difference **(D)** qRT-PCR was used to detect the expression of PANDAR in thyroid gland carcinoma tissues and adjacent tissues (30 patients in total). It was shown that PANDAR was highly expressed in cancer tissues compared with adjacent tissues, and the difference was statistically significant. **(E)** qRT-PCR was used to detect the expression of PANDAR in seven cell lines (Nthy-ori3-1, K1, TPC-1, K1, FTC133, XTC-1, B-CPAP), and the expression of PANDAR was significantly higher than the normal cell line Nthy-ori 3-1.

We used sequencing results from 10 pairs of thyroid gland carcinoma and adjacent tissues for bioinformatics analysis. First, PCA analysis showed the difference between the cancer and adjacent groups (Figure 1A). A heat map was generated showing all non-coding RNAs differentially expressed between the cancer and adjacent groups (Figure 1B). Volcano plot analysis of the differentially expressed non-coding RNAs showed that lncRNA-PANDAR is highly expressed and miR-637 is expressed at low levels in thyroid gland carcinoma compared to the adjacent group. To investigate the role of lncRNA-PANDAR in the pathogenesis of thyroid gland carcinoma, qRT-PCR was used to detect the expression of PANDAR in thyroid gland carcinoma tissues (30 patients in table 1) and 7 cell lines. The results showed that the relative expression of PANDAR mRNA was significantly higher in thyroid carcinoma tissues than in adjacent normal tissues (Figure 1D, P < 0.001). Interestingly, the relative expression of PANDAR in the seven thyroid gland carcinoma cell lines (Nthy-ori3-1, K1, TPC-1, K1, FTC133, XTC-1, B-CPAP) was significantly higher than that of the normal cell line Nthy-ori3-1 (Figure 1E, P < 0.01).

### Effects of PANDAR knockdown on the proliferation and invasion of TPC-1 and K1 cells *in vitro*

**Figure 2:**
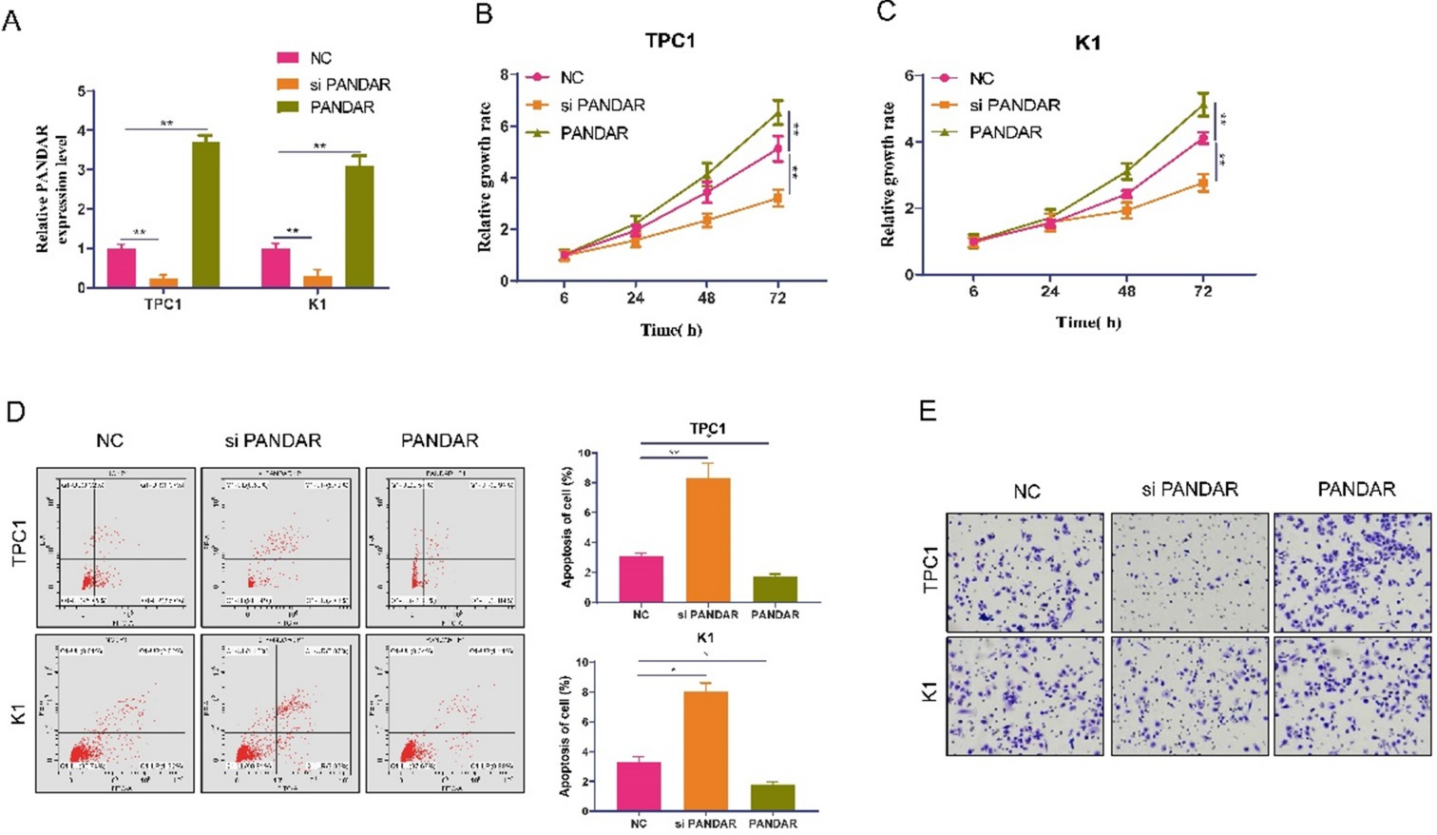
** Effect of lncRNA-PANDAR on apoptosis, viability, and migration of thyroid gland carcinoma cells. (A)** Validation of lncRNA-PANDAR overexpression or knockdown in K1 and TPC-1 cells was determined by qRT-PCR. **(B and C)** MTT proliferation assay for lncRNA-PANDAR overexpression or knockdown in K1 and TPC-1 cells. **(D)** Flow cytometric analysis of apoptosis overexpressing or knocking down K1 and TPC-1. **(E)** Transwell invasion assay for lncRNA-PANDAR overexpression or knockdown in K1 and TPC-1 cells. lncRNA-PANDAR significantly promote the invasion of thyroid cancer. SiPANDAR: PANDAR siRNA, PANDAR: PANDAR overexpression, NC: negative control. * P <0.05, ** P <0.01.

As shown in Figure 1E, the expression of PANDAR in TPC-1 and K1 cells was relatively higher than that in the other three cell lines. Therefore, we selected the TPC-1 and K1 cell lines as models to study the effects of PANDAR on cell proliferation and apoptosis. We knocked down PANDAR expression in TPC-1 and K1 cancer cells by transfection with NC-siRNAs or si-PANDAR. As shown in Figure 2A, qRT-PCR demonstrated that cells transfected with si-PANDAR showed a significant decrease in PANDAR mRNA expression levels compared to the NC groups in both cell lines (P < 0.05; Figure 2A). To determine the effect of PANDAR on the viability and proliferation of thyroid gland carcinoma cells *in vitro*, MTT assays were performed, which showed that PANDAR knockdown significantly inhibited the viability of TPC-1 and K1 cells in a time-dependent manner (P < 0.05; Figure 2B-C). The ability of TPC-1 and K1 cells to undergo apoptosis was also significantly enhanced after knocking down PANDAR compared to that in the negative control (P < 0.01; Figure 2D). These results indicate that PANDAR expression has a significant promoting effect on the growth of thyroid gland carcinoma cells. Furthermore, knockdown of PANDAR resulted in attenuated invasion of TC cells as measured by the transwell invasion assay (Figure 2E).

### PANDAR can regulate the progression of thyroid gland carcinoma by targeting miR-637

**Figure 3:**
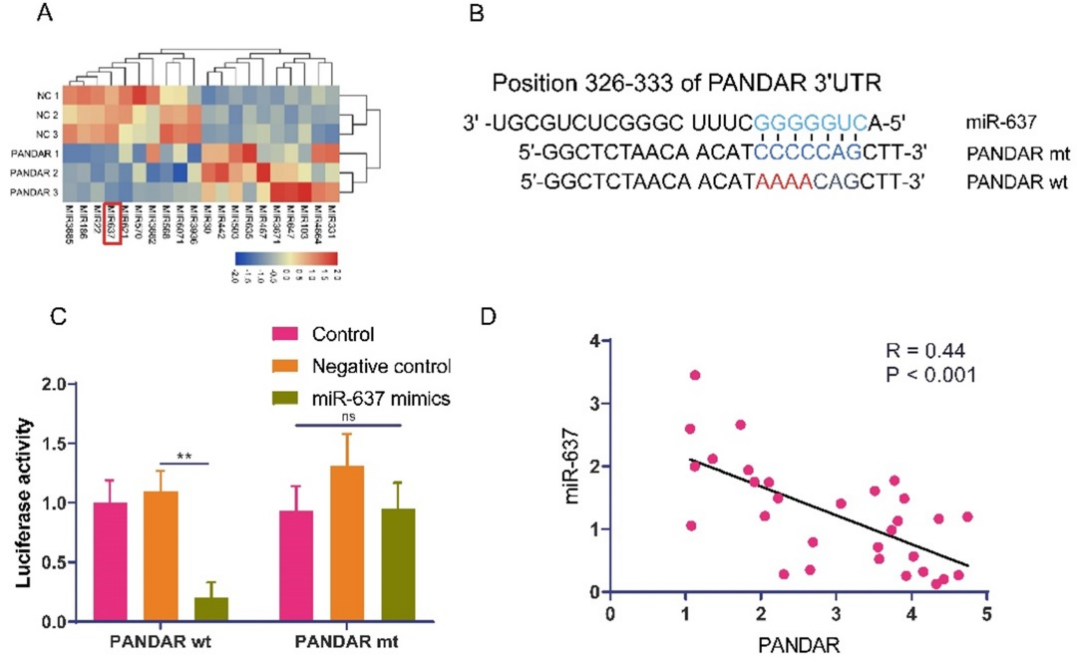
** LncRNA-PANDAR is negatively correlated with miR-637. (A)** Sequencing PANDAR overexpressing and negative control cells. The miR-637 had the lowest expression, the most significant difference. **(B)** Predicted binding site for 3'UTR of miR-637 and PANDAR sequences. **(C)** Luciferase reporter assay for miR-637 that directly targets PANDAR. **(D)** A negative correlation between the expression of PANDAR and miR-637 in thyroid gland carcinoma patients. * P <0.05, *** P <0.01.

To investigate how PANDAR regulates proliferation and metastasis in thyroid gland carcinoma, we transfected the PANDAR plasmid into TPC-1 cells for 72 hours and performed transcriptome sequencing analysis of the differentially expressed downstream non-coding RNAs of PANDAR. From the sequencing analysis, the heat map showed that the expression of miR-637 was significantly downregulated in the PANDAR overexpression group (Figure 3A). Then, we searched for a target gene candidate of PANDAR (mirdb.org) and identified miR-637 as a candidate target of PANDAR (Figure 3B). Therefore, we further studied miR-637. To investigate whether PANDAR is involved in the regulation of miR-637, the luciferase reporter was performed, which confirmed that PANDAR directly targets the 3'UTR of miR-637 (Figure 3C). Consistent with the *in vitro* results, the clinical sample analysis results also showed an inverse association between PANDAR and miR-637 in 30 thyroid gland carcinoma specimens (Figure 3D). Together, these findings indicate that PANDAR inhibits miR-637 expression by directly targeting its 3'UTR.

**Figure 4:**
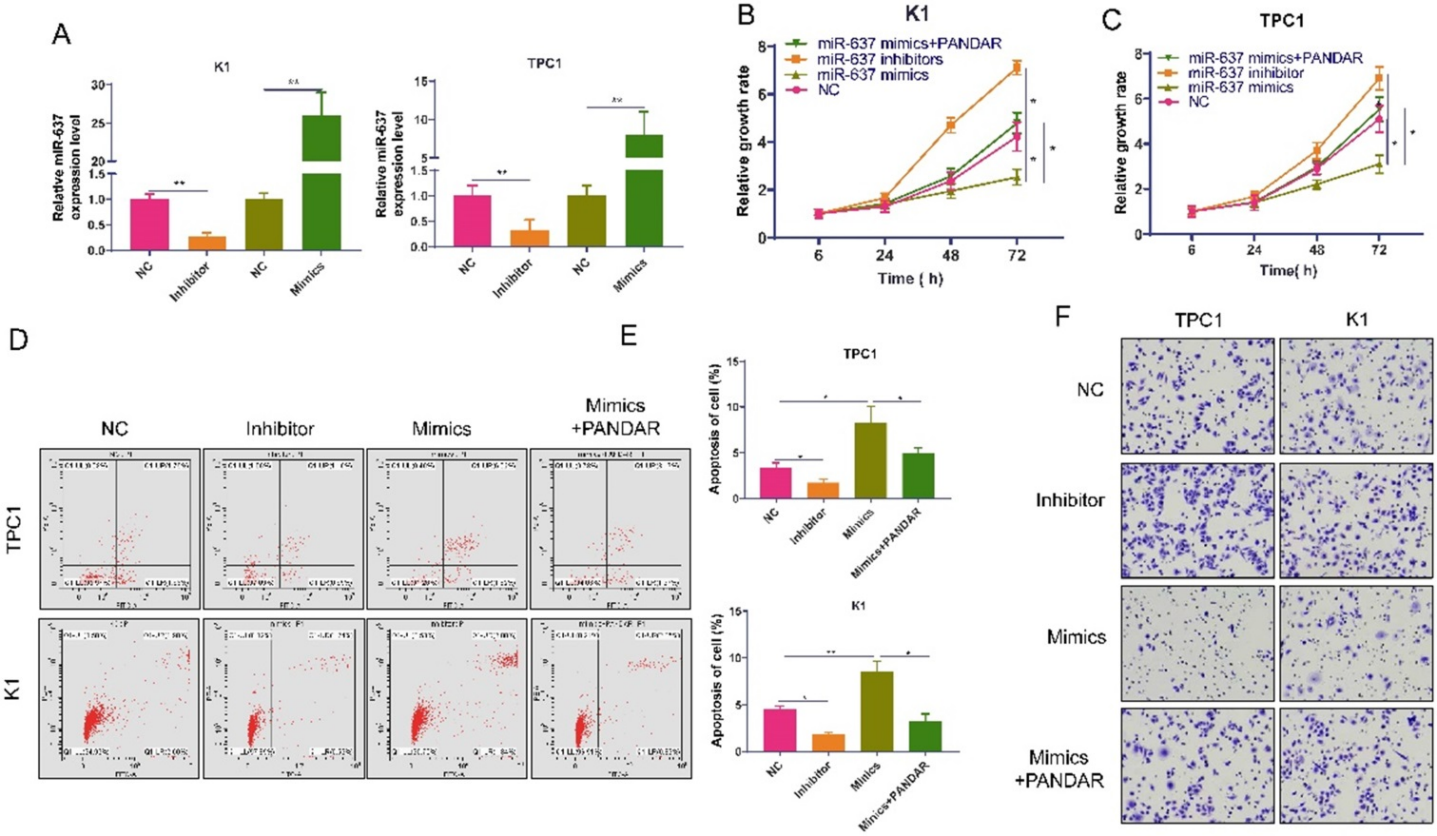
** Overexpression of MiR-637 inhibits the progression of thyroid gland carcinoma. (A)** Validation of miR-637 mimics or inhibitor in K1 and TPC-1 cells as determined by qRT-PCR. **(B and C)** miR-637 mimics/inhibitor and PANDAR overexpressed MTT proliferation assays of transfected K1 and TPC-1 cells. **(D)** Flow cytometry analysis of apoptosis in K1 and TPC-1 cells transfected with miR-637 mimics/inhibitor and PANDAR overexpression. **(E)** Statistical analysis of apoptosis. **(F)** Transwell invasion assay in K1 and TPC-1 cells transfected with miR-637 mimetic\inhibitor and lncRNA-PANDAR overexpression. * P <0.05, ** P <0.01.

We transfected miR-637-specific inhibitors or mimics into K1 and TPC-1 cells and detected miR-637 expression levels 72 hours after transfection (Figure 4A). MiR-637 inhibitors promoted thyroid gland carcinoma cell proliferation, while miR-637 mimics inhibited thyroid gland carcinoma cell proliferation, and these processes could be reversed by PANDAR (Figure 4B, C). Apoptosis test results also showed the same trend (Figure 4D and E). The apoptotic rate was higher when miR-637 was overexpressed, and it was lower when miR-637 was inhibited. MiR-637 inhibitors promoted thyroid gland carcinoma cell migration, while miR-637 mimics inhibited thyroid gland carcinoma cell migration, and these processes could be reversed by PANDAR (Figure 4F).

### MiR-637 can directly target KLK4 in thyroid gland carcinoma

**Figure 5:**
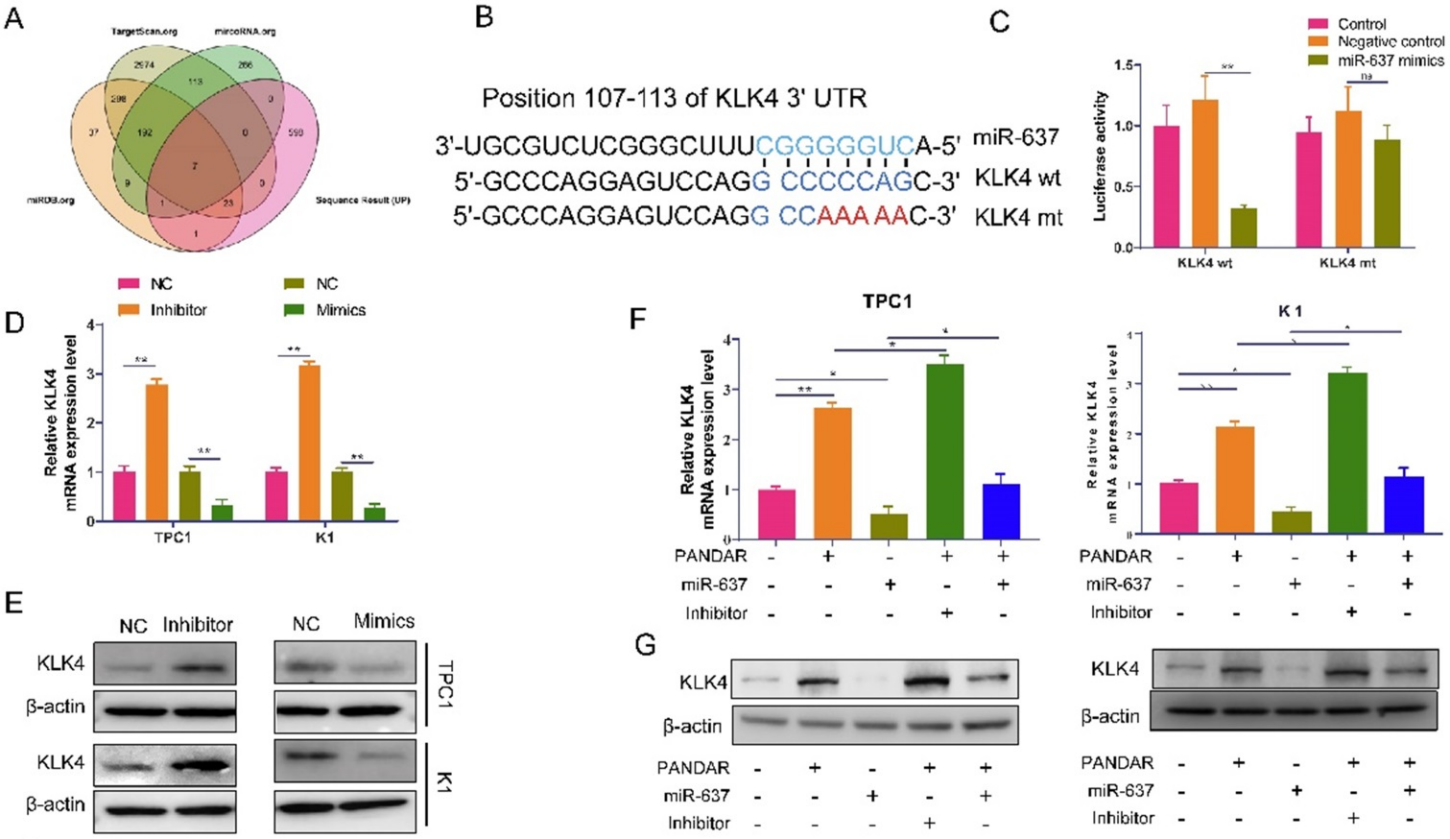
** MiR-637 negatively correlated with KLK4 was highly expressed thyroid gland carcinoma. (A)** We used TargetScan.org, miRDB.org, microRNA.org and our transcriptome sequencing results analysis the target of miR-637. **(B)** We have predicted the direct target relationship between miR-637 and KLK4 by TargetScan. **(C)** Luciferase reporter assay analysis the MiR-637 directly targeted at KLK4 3'UTR. **(D)** Expression of KLK4 in miR-637 mimics or inhibitors in K1 and TPC-1 cells as determined by qRT-PCR. **(E)** Expression of KLK4 when transfected with miR-637 mimics or inhibitors in K1 and TPC-1 cells as determined by Western blot. **(F)** Expression of KLK4 mRNA when transfected with miR-637 mimics or inhibitors and PANDAR exist or not in K1 and TPC-1 cells as determined by qRT-PCR. **(G)** Expression of KLK4 protein when transfected with miR-637 mimics or inhibitors and PANDAR exist or not in K1 and TPC-1 cells as determined by Western blot. *P<0.05, ** P<0.001.

To investigate how miR-637 regulates proliferation and metastasis in thyroid gland carcinoma, we used TargetScan.org, miRDB.org, microRNA.org, and our transcriptome sequencing results to identify the target of miR-637. From the analysis, the Venn diagram showed that there were a total of 7 genes that were significantly different in all databases (Figure 5A). The expression of KLK4 was the most significantly different, and then, TargetScan predicted that miR-637 may directly target KLK4 (Figure 5B). Therefore, we investigated whether miR-637 regulated KLK4, and the luciferase reporter assay confirmed that miR-637 directly targets the 3'UTR of KLK4, as demonstrated by the significantly lower luciferase activity in the miR-637 mimics+KLK4-wt group (Figure 5C). The relationship was also confirmed in pancreatic cancer cells by qPCR (Figure 5D) and Western blot analysis (Figure 5E). Overall, these findings indicate that miR-637 inhibited KLK4 expression by directly targeting its 3'UTR. We also tested the relationship between PANDAR and KLK4. As shown in Figure 5F and 5G, not only could miRNA-637 affect the expression of KLK4, but PANDAR also affected the expression of KLK4, and upregulation of miR-637 could block the expression of KLK4 induced by PANDAR at both the mRNA (Figure 5F) and protein (Figure 5G) levels. PANDAR could regulate KLK4 expression by targeting miR-637.

### KLK4 can suppress thyroid gland carcinoma cell progression

**Figure 6:**
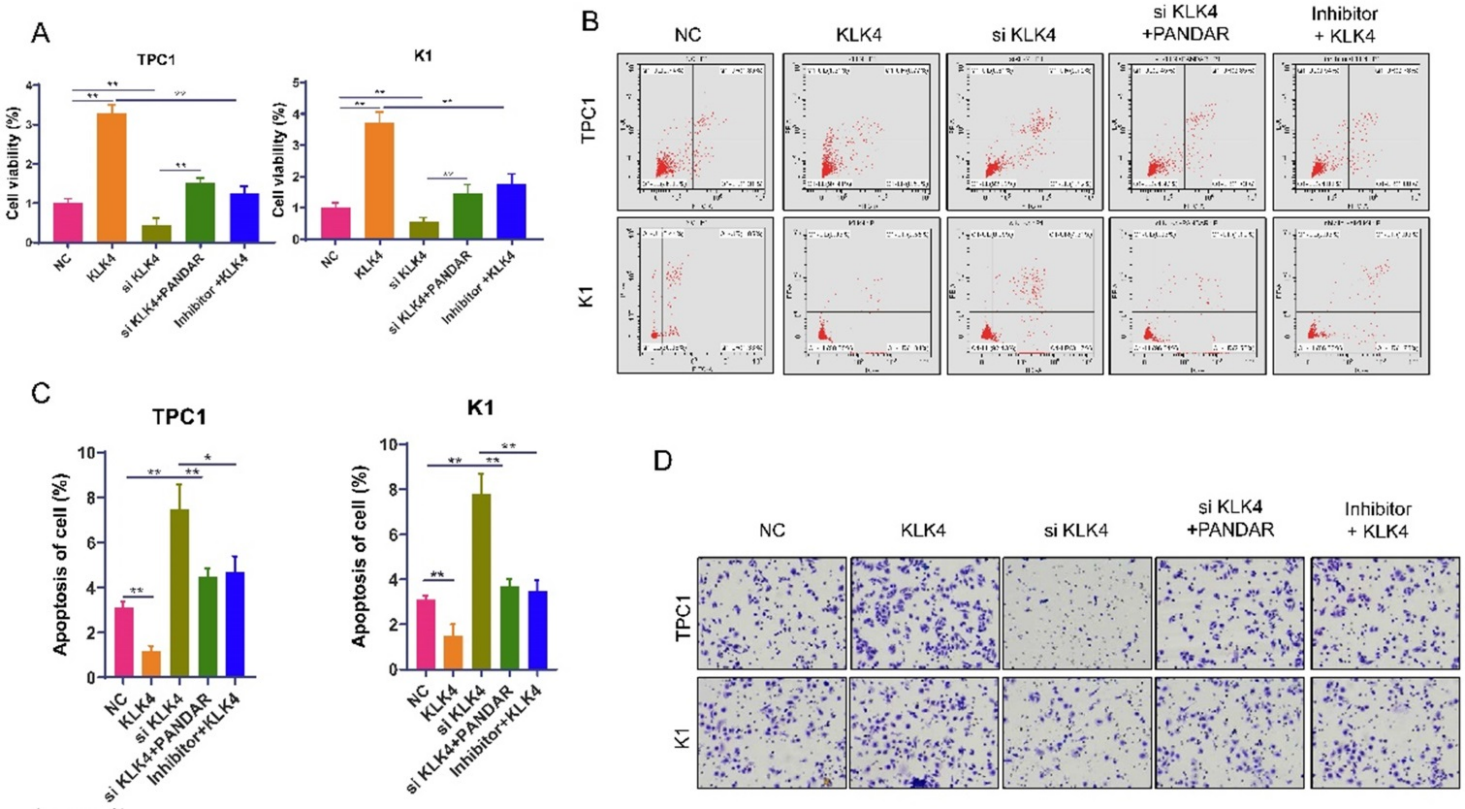
** KLK4 could suppress thyroid gland carcinoma cells progress. (A)** MTT proliferation assay of specified plasmid or RNA transfection transfected in K1 and TPC-1 cells. **(B)** Flow cytometric analysis of apoptosis when transfected with KLK4 plasmid, siRNA, miR-637 inhibitors or PANDAR, respectively in K1 and TPC-1 cells in K1 and TPC-1 cells. **(C)** Statistical analysis of apoptosis. **(D)** Transwell invasion assay when transfected with KLK4 plasmid, siRNA, miR-637 inhibitors or PANDAR, respectively in K1 and TPC-1 cells. *P<0.05, ** P<0.01.

Next, we use MTT assays to show that thyroid gland carcinoma cell proliferation was inhibited in the siKLK4 group but enhanced in the KLK4 overexpression group. PANDAR could reverse the growth inhibition of thyroid gland carcinoma cells caused by KLK4 downregulation to some extent. The miR-637 inhibitor reversed thyroid gland carcinoma cell proliferation caused by KLK4 overexpression (Figure 6A). The apoptosis assay displayed similar changes in cell apoptosis. The cell apoptosis rate was much lower in the KLK4 overexpression group, but in the siKLK4 group, it was higher, which could be reversed by PANDAR and miR-637 inhibitors (Figure 6B, C). Conversely, cell migration in the KLK4 group was promoted, while migration in the siKLK4 group was suppressed, and these effects could be reversed by PANDAR and miR-637 inhibitors (Figure 6D).

### PANDAR overexpression promotes thyroid gland carcinoma progression *in vivo*

**Figure 7:**
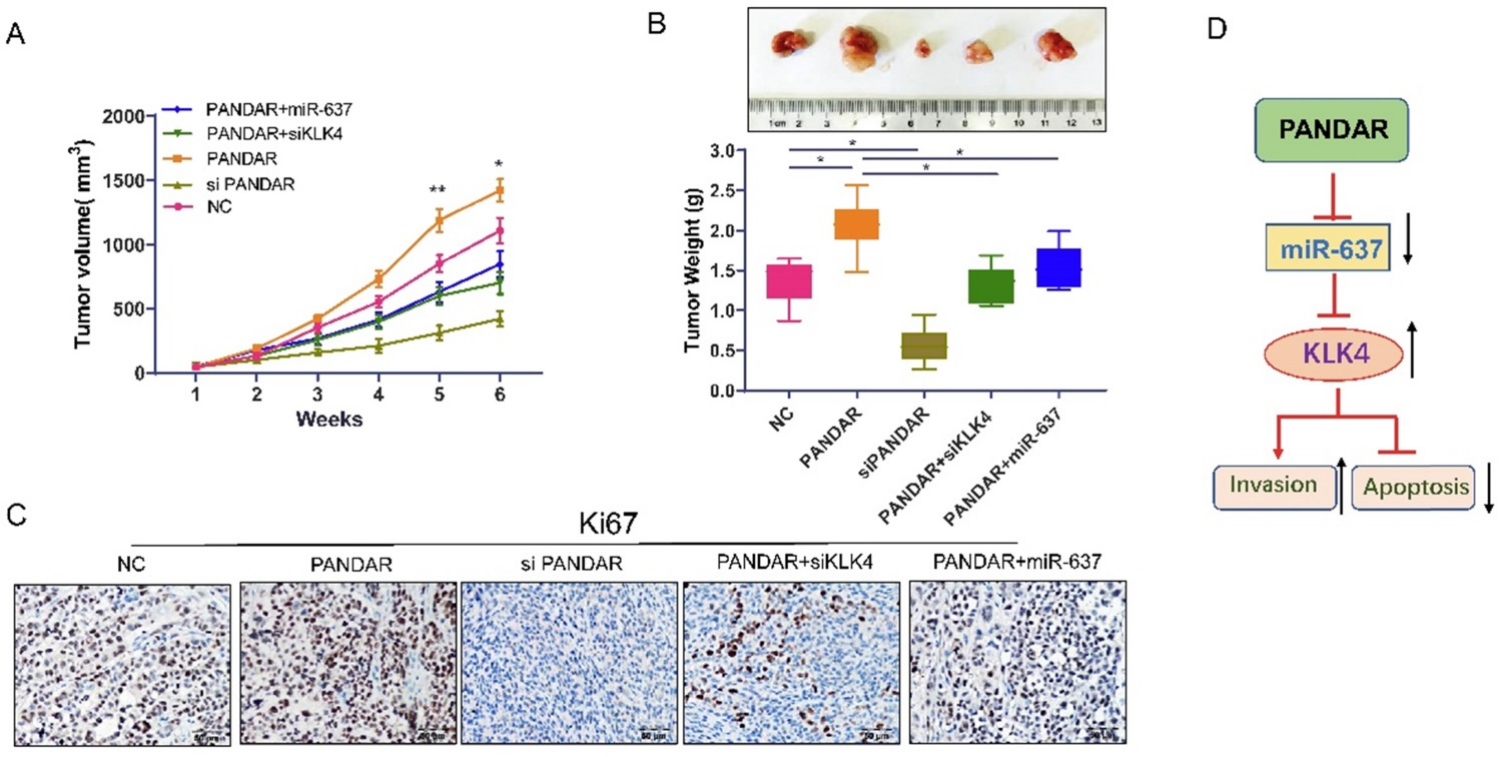
** Suppress the LncRNA PANDAR expression inhibited caner progression in nude mice. (A)** Tumour volume changes after transfected with PANDAR, siPANDAR, PANDAR+miR-637 mimics or PANDAR+siKLK4 in TPC-1 cells xenograft tumor, Volume calculation formula: (length x width2)/2. **(B)** Tumor weight changes when transfected with PANDAR, siPANDAR, PANDAR+miR-637 mimics or PANDAR+siKLK4. **(C)** Ki-67 IHC assay results of the xeograft tumor tissue, the tumor proliferation induced by PANDAR can be block through the miR-637 mimics and siKLK4. **(D)** Mechanism model of LncRNA-PANDAR regulating the progression of thyroid carcinoma via targeting miR-637/KLK4. *P<0.05, **P<0.01.

As shown in Figure 7A and 7B, tumour volume and tumour weight were significantly increased when PANDAR was overexpressed. The tumour growth induced by PANDAR could be reversed by siKLK4 and miR-637 mimics. Consistent with these results, Ki-67 IHC assay (Figure 7C) results clearly showed that the tumour proliferation induced by PANDAR could be blocked by miR-637 mimics and siKLK4 *in vivo*.

## Discussion

Recently, there has been increasing evidence that lncRNAs play an important regulatory role in the development of various types of cancer 8, 23. Many papers have shown that PANDAR is abnormally expressed in various cancers 10, 24, 25. However, few studies have reported the potential role of lincRNA PANDAR in thyroid gland carcinoma. In this study, we found that PANDAR is highly expressed in thyroid gland carcinoma tissues and cell lines. In addition, loss-of-function assays showed that knockdown of PANDAR significantly inhibited proliferation and invasion. In addition, lncRNA PANDAR induces apoptosis in thyroid gland carcinoma cells 26.

In this study, we clarified the anti-thyroid gland carcinoma mechanism of PANDAR. The data showed that knocking down PANDAR expression significantly inhibited thyroid gland carcinoma cell proliferation and colony formation, suggesting that PANDAR can promote thyroid gland carcinoma cell proliferation. These results were consistent with our previous studies 26. Moreover, we searched for candidate microRNA targets of PANDAR (mirdb.org) and identified miR-637 as a candidate target of PANDAR. The luciferase reporter assay also showed that lincRNA PANDAR can target miR-637, and PANDAR can affect the proliferation and metastasis of thyroid tumours by regulating miR-637. Previous studies have shown that KLK4 can promote tumour proliferation, migration and invasion in ovarian cancer 27. Here, we used a series of *in vitro* experiments to identify KLK4 as a target gene of miR-637 in TC. Our data showed that KLK4 expression was increased or downregulated in TC cells by the inhibition or ectopic expression of miR-637, respectively. In addition, a luciferase reporter assay showed that miR-637 directly targeted the 3'UTR of KLK4. Additionally, our data demonstrated that restoring miR-637 expression blocked the increase in KLK4 induced by PANDAR overexpression and that miR-637 promoted metastasis and proliferation. Our data are consistent with Zhang et al.'s report 27. Cui et al. reported that the apoptosis of OSCC cells was enhanced by KLK4 silencing and that KLK4 regulated apoptosis-related proteins and suppressed apoptosis 28. Therefore, we infer that PANDAR regulates KLK4 through miR-367 to affect tumour apoptosis and metastasis. We will continue to explore this hypothesis in future research. Taken together, these data suggest that PANDAR inhibits TC metastasis and proliferation through inhibition of miR-637 by targeting KLK4 (Figure 7D).

Taken together, our results indicate that downregulation of PANDAR expression inhibits cell proliferation and metastasis. Therefore, PANDAR may be a promising therapeutic target and a novel molecular biomarker for TC.

## Figures and Tables

**Table 1:** Characteristics of thyroid carcinoma patients

Characteristics	Variable	Number (%)
Age (years)	Range (means±SD)	35-62 (50±8)
Gender	Male	14 (46.7)
	Female	16 (53.3)
Family history	No	25 (16.7)
	Yes	5 (83.3)
Pathological type	Papillary carcinoma	30 (100)
